# Comments on: Is Pethidine Safe during Labor? Systematic Review

**DOI:** 10.1055/s-0038-1675201

**Published:** 2018-10

**Authors:** Tatiana Henriques Leite, Emanuele Souza Marques, Vitor Barreto Paravidino

**Affiliations:** 1 Social Medicine Institute, Universidade do Estado do Rio de Janeiro, Maracanã, RJ, Brazil

Dear Editor,

Several studies demonstrate that pethidine is safe and effective for the parturient;^1^ however, its use has been widely questioned because of the possible side effects on the fetus. Nunes et al (2017)^2^ conducted a systematic review to determine if pethidine during labor is safe for the conceptus; however, some important limitations of this review should be discussed.

The main issue is related to the search strategy. According to the Cochrane Handbook for Systematic Reviews,^3^ three databases should be considered for a clinical trial search: Medline, Embase and CENTRAL. In addition, in Brazil, the Ministry of Health recommends that a systematic review should include, at least, four essential databases (Medline, Embase, CENTRAL and Lilacs) and an area-specific one.^4^ Nevertheless, Nunes et al^2^ described that only two databases (Medline and Virtual Health Library Biblioteca Virtual em Saúde [BVS, in the Portuguese acronym]) were used.

The authors reported the search was performed using the keywords *pethidine in labor*, alone or combined with each other; however, this search strategy is incorrect. Searches should be conducted using Medical Subject Headings (MeSh terms) and Boolean operators. According to the literature, there are several pethidine synonymous (meperidine, piperosal, dolosal, demerol and dolantine), and these terms were not considered.

Combining these two limitations mentioned above and the same inclusion/exclusion criteria used by the authors, at least eight eligible articles were not included in this review.^5^ ^6^ ^7^ ^8^ ^9^ ^10^ ^11^ ^12^ This number represents an underestimation of, at least, 50% of all papers included in this systematic review. In addition, the search strategy was limited to a 16-year period. This approach ignores all evidence produced before the considered period and, according to the Cochrane Handbook, this is not indicated.^3^

Another important issue regards the lack of a specific question structured by the population, intervention, control, outcome (PICO) process. It is not clear to the readers who the participants [P] were (nulliparous, multiparous, high risk pregnancy); the intervention [I] (drug concentration, drug administered intravenously or intramuscularly, combined or not with other medications, at which labor stage the intervention was applied); the control group [C] (placebo, other drugs) and how the outcome was assessed [O]. All these characteristics could influence the result of the review. Therefore, this information should be collected and presented in a subgroup analysis.

Other important limitations should be highlighted: (1) the authors did not perform a risk of bias assessment. This is the only available strategy to evaluate internal validity—an important criterion in epidemiologic studies; (2) the authors did not describe the systematic review following the preferred reporting items for systematic reviews and meta-analyses (PRISMA) criteria.^13^ Study selection, data collection process, flowchart and other aforementioned parameters were not present as recommended; (3) the authors reported that “sample size bellow 25 patients” is an exclusion criterion because they are more prone to erroneous conclusions. However, this statement is incorrect. Actually, a sample size is specific for a given study and 25 patients or less can be adequate, considering the parameters used.

In conclusion, although the authors of this review analyzed an important question, the limitations and methodological errors mentioned above might have influenced the results. Therefore, any conclusion or recommendation concerning the results of this review should be interpreted with caution. Future systematic reviews and meta-analyses should perform an exhaustive search of the literature and use an appropriate methodological approach.

## Notes

Authors' Reply Dear Editor, The authors have nothing to declare about the letter to editor.
